# Weight Loss Improved Hypothalamic GH Deficiency but not Hypogonadotropic Hypogonadism in a Man With Down Syndrome

**DOI:** 10.1210/jcemcr/luae114

**Published:** 2024-07-18

**Authors:** Yukie Nakagawa, Katsumi Taki

**Affiliations:** Department of Endocrinology and Metabolism, Fujiyoshida Municipal Medical Center, Fijiyoshida City, Yamanashi 403-0032, Japan; Department of Endocrinology and Metabolism, Fujiyoshida Municipal Medical Center, Fijiyoshida City, Yamanashi 403-0032, Japan

**Keywords:** Down syndrome, severe obesity, hypogonadotropic hypogonadism, hypothalamic growth hormone deficiency, leptin

## Abstract

Down syndrome (DS) is associated with several endocrine disorders, including diabetes, obesity, and primary hypogonadism. Here, we present a man with DS who manifested with atypical hypogonadotropic hypogonadism and in whom weight loss resulted in the improvement of hypothalamic GH deficiency. A 27-year-old man with DS and severe obesity was admitted for hypoxia resulting from obesity hypoventilation syndrome. Laboratory tests showed normal levels of LH and FSH despite low testosterone and free testosterone levels. Moreover, thyroid stimulating hormone and prolactin levels were slightly elevated, although a euthyroid function was observed, and GH and IGF-1 levels were low. Endocrinological stimulation tests revealed hypogonadotropic hypogonadism and hypothalamic GH deficiency. Reduction in body weight by 35.3% resulted in the improvement of the IGF-1, thyroid stimulating hormone, and prolactin levels to the reference range, whereas the LH and FSH levels remained low, despite slight elevation. Levels of leptin, which suppresses the hypothalamus–gonadotroph–gonadal axis and upregulates thyrotropin-releasing hormone expression, decreased with weight loss. Furthermore, ghrelin, whose levels increase with weight loss, stimulates GH secretion. Thus, leptin and ghrelin could have contributed to the observed changes in the pituitary hormone profile after weight loss.

## Introduction

Down syndrome (DS), the most common chromosomal abnormality known as trisomy of chromosome 21, is characterized by intellectual disability, congenital heart disease, obstructive sleep apnea, thyroid dysfunction, leukemia, diabetes, short stature, infertility, and obesity. Women with DS are mostly fertile, whereas men with DS exhibit fertility issues because of primary hypogonadism (1).

Obesity results in decreased GH and IGF-1 secretion (2), and it is more severe in patients with DS than in the general population (3). Reports indicate that patients with DS may have hypothalamic GH deficiency (GHD), which results from an impaired GH-releasing hormone (GHRH)–GH–IGF-1 axis (3). However, changes in hypothalamic GHD in adults with DS following weight loss remain unknown.

Here, we present a patient with DS and severe obesity who manifested with hypogonadotropic hypogonadism and hypothalamic GHD and in whom weight loss improved the secretion of anterior pituitary hormones, particularly GH, but not gonadotropic hormones.

## Case Presentation

A 27-year-old man with congenital heart disease was diagnosed with DS, characterized by trisomy of chromosome 21. He was previously diagnosed with leukemia at the age of 1 year, which went into complete remission following chemotherapy. His weight gradually increased, reaching 60 kg (body mass index [BMI] 27.4 kg/m^2^) by the age of 20 years. In recent years, his weight has been maintained at between 80 and 90 kg (BMI, 36.5 kg/m^2^–41 kg/m^2^).

A few years before admission, he was diagnosed with sleep apnea and received continuous positive airway pressure, which he discontinued after a year. Comorbidities included ventricular septal defect, type 2 diabetes, hyperuricemia, and bronchial asthma. His medications included metformin, dapagliflozin, dulaglutide, febuxostat, and a dry powder inhaler (fluticasone, vilanterol, umeclidinium). A month before admission, he rapidly gained 20 kg, resulting in respiratory distress.

## Diagnostic Assessment

On admission, his height and weight were 148 cm and 115.9 kg, respectively (BMI, 52.9 kg/m^2^). Physical examination revealed severe obesity, slight indurating edema on both lower legs, and no olfactory abnormalities. Axillary and pubic hair were absent, and no secondary sexual characteristics, such as a testicular volume of 1.06 mL, which is typical for a 10-year-old boy (4), were observed. His blood pressure was 116/82 mm Hg, and his pulse rate was 102 beats/min. Oxygen saturation was 83% on room air, indicating tachycardia and hypoxia.

Arterial blood gas values on room air suggested respiratory acidosis resulting from chronic obesity hypoventilation syndrome (Table 1). Despite consumption of several antidiabetic drugs, he had elevated hemoglobin A1c (HbA1c) levels (Table 2). Brain natriuretic peptide concentrations were elevated as well (Table 2), indicating cardiac overload.

**Table 1.** Arterial blood gas values on room air

	Arterial blood gas values	Reference range
	Conventional units (SI units)	Conventional units (SI units)
pH	**7.283** **(7.283)**	7.35-7.45 (7.35-7.45)
pCO_2_	**64.6 mmHg** **(8.59 kPa)**	35-45 mmHg (4.66-5.99 kPa)
pO_2_	78.5 mmHg (10.44 kPa)	75-100 mmHg (9.98-13.3 kPa)
Bicarbonate	**29.9 mmol/L** **(29.9 mmol/L)**	20-26 mmol/L (20-26 mmol/L)
Lactate	1.1 mmol/L (1.1 mmol/L)	0.5-1.98 mmol/L (0.5-1.98 mmol/L)

Results out of the reference range are shown in bold.

Abbreviations: pCO_2_, arterial carbon dioxide tension; pO_2_, arterial oxygen tension.

**Table 2.** General laboratory data

	Laboratory data	Reference range
	Conventional units (SI units)	Conventional units (SI units)
TP	7.2 g/dL (72.0 g/L)	6.6-8.1 g/dL (66.0-81.0 g/L)
Albumin	**3.2 g/dL** **(32 g/L)**	4.1-5.1 g/dL (41.0-51.0 g/L)
T-Bil	0.62 mg/dL (10.6 µmol/L)	0.4-1.5 g/dL (6.84-25.66 µmol/L)
LDH	203 U/L (3.39 µkat/L)	124-222 U/L (2.07-3.71 µkat/L)
TG	128 mg/dL (1.45 mmol/L)	40-234 mg/dL (0.45-2.64 mmol/L)
LDL-chol	117 mg/dL (3.03 mmol/L)	65-163 mg/dL (1.68-4.22 mmol/L)
HDL-chol	52 mg/dL (1.35 mmol/L)	38-90 mg/dL (0.98-2.33 mmol/L)
AST	**43 U/L** **(0.72 µkat/L)**	13-30 U/L (0.22-0.50 µkat/L)
ALT	**47 U/L** **(0.78 µkat/L)**	10-42 U/L (0.17-0.70 µkat/L)
ɤ-GT	**199 U/L** **(3.32 µkat/L)**	13-64 U/L (0.22-1.07 µkat/L)
ALP	57 U/L (0.95 µkat/L)	38-113 U/L (0.63-1.89 µkat/L)
BUN	12.8 mg/dL (4.57 mmol/L)	8.0-20.0 mg/dL (2.86-7.14 mmol/L)
Creatine	0.68 mg/dL (51.85 µmol/L)	0.65-1.07 mg/dL (49.56—81.59 µmol/L)
Uric acid	**8.4 mg/dL** **(0.50 mmol/L)**	3.7-7.8 mg/dL (0.22-0.46 mmol/L)
Sodium	141 mmol/L (141 mmol/L)	138-145 mmol/L (138-145 mmol/L)
Potassium	4.3 mmol/L (4.3 mmol/L)	3.6-4.8 mmol/L (3.6-4.8 mmol/L)
CRP	**2.25 mg/dL** **(22.5 mg/L)**	<0.14 mg/dL (<1.4 mg/L)
BNP	**91.2 pg/mL** **(91.2 ng/L)**	≤18.4 pg/mL (≤18.4 ng/L)
Glucose	**200 mg/dL** **(11.1 mmol/L)**	73-109 mg/dL (4.05-6.05 mmol/L)
HbA1c	**9.4%** **(0.094 proportion of total hemoglobin)**	4.9-6.0% (0.049-0.06 proportion of total hemoglobin)
WBC	5420/µL (5.42 × 10^9^ /L)	3300-8600/µL (3.30-8.60 × 10^9^ /L)
Hb	**18.7 g/dL** **(187 g/L)**	13.7-16.8 g/dL (137-168 g/L)
Hct	**59.4%** **(0.594 proportion of 1.0)**	40.7-50.1% (0.407-0.501 proportion of 1.0)
MCV	90.8 fL (90.8 fL)	83.6-98.2 fL (83.6-98.2 fL)
Plt	191 × 10^3^/µL (191 × 10^9^ /L)	158-348 × 10^3^/µL (158-348 × 10^9^/L)
D-dimer	**2.5 µg/mL** **(13.69 nmol/L)**	0-1.0 µg/mL (0-5.48 nmol/L)

Results out of the reference range are shown in bold.

Abbreviations: TP, total protein; T-Bil, total bilirubin; LDH, lactate dehydrogenase; TG, triglycerides; LDL-chol, low-density cholesterol; HDL-chol, high-density cholesterol; AST, aspartate aminotransferase; ALT, alanine aminotransferase; ɤ-GT, γ-glutamyl transferase; ALP, alkaline phosphatase; BUN, urea nitrogen; CRP, C-reactive protein; BNP, brain-type natriuretic peptide; HbA1c, hemoglobin A1c (glycated hemoglobin); WBC, white blood cell count; Hb, hemoglobin; Hct, hematocrit; MCV, mean corpuscular volume; Plt, Platelet count (thrombocytes).

Results of endocrinological evaluations are presented in Table 3. Leptin levels were elevated (Table 3). Although free triiodothyronine and free thyroxine levels were within their respective ranges, TSH levels were elevated and thyroid autoantibodies were negative (Table 3). Prolactin (PRL) levels were also mildly elevated. Despite normal GH levels, decreased IGF-1 levels (Table 3) suggested reduced GH secretion. Despite decreased testosterone (TT) and free TT levels, LH and FSH levels were within ranges (Table 3). Although primary hypogonadism is usually observed in patients with DS, our patient was diagnosed with GHD and secondary hypogonadism.

**Table 3.** Endocrinological laboratory tests

	Endocrinological laboratory data	Reference range
	Conventional units (SI units)	Conventional units (SI units)
Leptin	33.1 ng/mL (33.1 μg/L)	0.6-8.9 ng/mL (0.6-8.9 μg/L)
fT3	2.7 pg/mL (4.15 pmol/L)	1.71-3.71 pg/mL (2.63- 5.70 pmol/L)
fT4	0.99 ng/dL (12.74 pmol/L)	0.70-1.48 ng/dL (9.01-19.05 pmol/L)
TSH	8.2 μIU/mL (8.2 mIU/L)	0.35-4.94 μIU/mL (0.35-4.94 mIU/L)
PRL	20.18 ng/mL (20.18 μg/L)	3.58-12.78 ng/mL (3.58-12.78 μg/L)
GH	0.44 ng/mL (0.44 μg/L)	≤ 2.47 ng/mL (≤2.47 μg/L)
IGF-1	58 ng/mL (7.60 nmolL)	116-322 ng/mL (15.20-42.18 nmol/L)
TT	0.28 ng/mL (0.97 nmol/L)	1.92-8.84 ng/mL (6.66-30.65 nmol/L)
FT	1.10 pg/mL (3.81 pmol/L)	7.6-23.8 pg/mL (26.35-82.52 pmol/L)
LH	1.34 mIU/mL (1.34 IU/L)	0.79-5.72 mIU/mL (0.79-5.72 IU/L)
FSH	2.21 mIU/mL (2.21 IU/L)	2.00-8.30 mIU/mL (2.00-8.30 IU/L)
ACTH	55.0 pg/mL (12.1 pmol/L)	7.2-63.3 pg/mL (1.58-13.93 pmol/L)
Cortisol	12.8 μg/dL (353.13 nmol/L)	3.7-19.4 μg/dL (102.08-535.21 nmol/L)
DHEA-S	120 μg/dL (3.24 μmol/L)	159-538 μg/dL (4.29-14.53 μmol/L)

Abbreviations: fT3, free triiodothyronine; fT4, free thyroxine; TSH, thyroid stimulating hormone; PRL, prolactin; GH, growth hormone; IGF-1, insulin-like growth factor-1; TT, testosterone; FT, free testosterone; LH, luteinizing hormone; FSH, follicle-stimulating hormone; ACTH, adrenocorticotropic hormone; DHEA-S, dehydroepiandrosterone sulfate.

**Figure 1.**
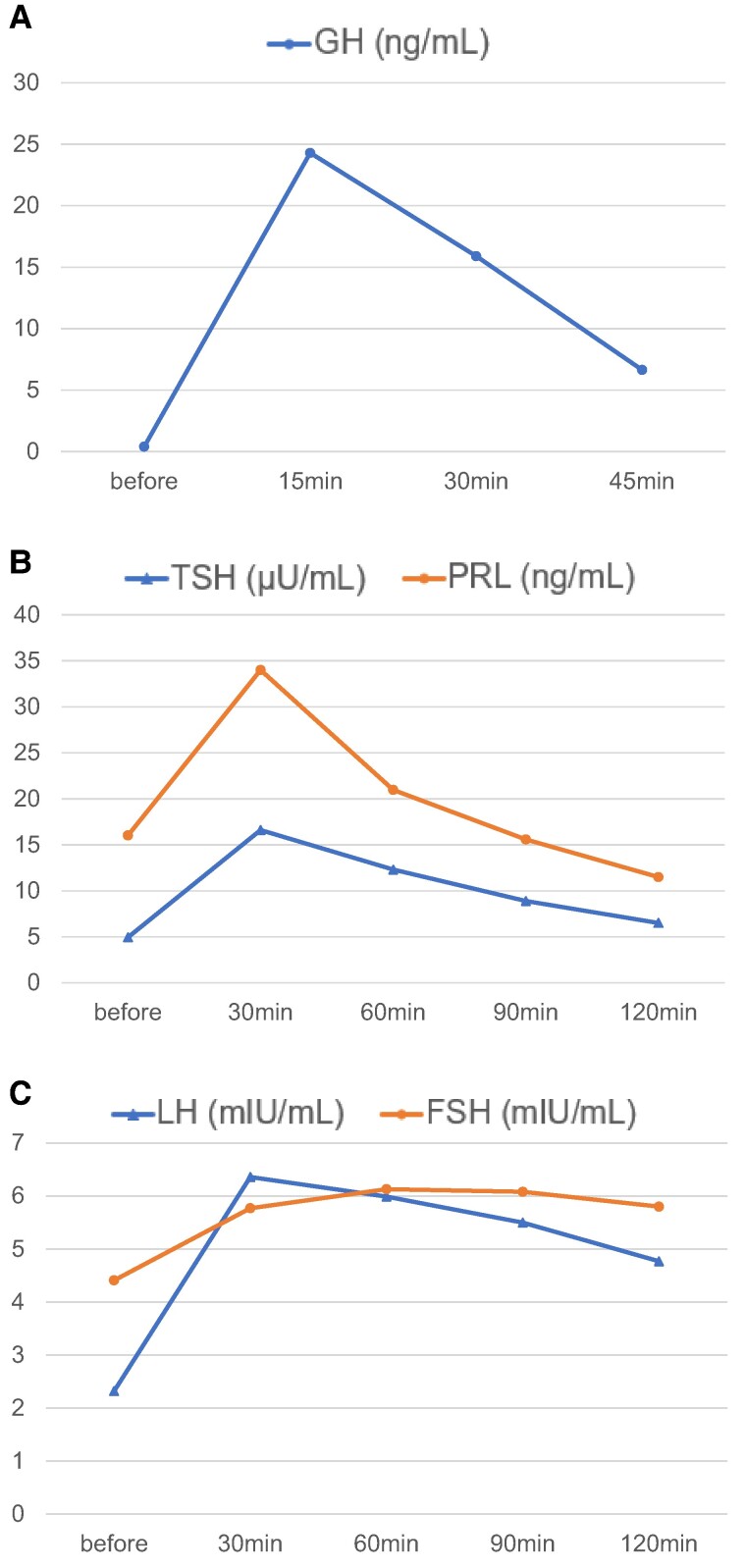
Endocrinological stimulation tests. (A) GH-releasing peptide-2 (GHRP-2) test. GH levels exhibited appropriate responses to GHRP-2. (B) Thyrotropin-releasing hormone (TRH) stimulation test. TSH and PRL levels exhibited appropriate responses to TRH. (C) GnRH stimulation test. LH and FSH levels exhibited impaired responses to GnRH. GH, TSH, PRL, LH, and FSH data can be converted to SI units by multiplying all values by 1.

To assess the etiology of GHD and hypogonadotropic hypogonadism, we performed endocrinological stimulation tests on admission days 17, 18, and 32 and pituitary magnetic resonance imaging on day 32, which revealed no neoplastic lesions in the pituitary gland, the pituitary stalk, or the hypothalamus. On day 17, the GH-releasing peptide-2 (GHRP-2) test, which involved an intravenous bolus injection of 0.1 mg of GHRP, revealed that GH levels increased to 24.3 ng/mL (24.3 μg/L) 15 minutes after the injection (Fig. 1A). Patients are generally diagnosed with pituitary GHD when peak GH levels are below 9 ng/mL (9 μg/L) on GHRP-2 test (5). Results indicated hypothalamic rather than pituitary cause of GHD. On day 18, the thyrotropin-releasing hormone (TRH) stimulation test, which involved an intravenous bolus injection of 0.5 mg of TRH, indicating that the baseline TSH and PRL levels (4.95 μIU/mL [4.95 mIU/L] and 16.02 ng/mL [16.02 μg/L], respectively) increased to their maximum levels (16.61 μIU/mL [16.61 mIU/L] and 34.08 ng/mL [34.08 μg/L], respectively) 30 minutes after the injection, showing appropriate reactivity to TRH (Fig. 1B). TRH stimulation test in normal individuals demonstrates peak TSH levels approximately 10 μIU/mL (10 mIU/L) within 15-30 minutes after the injection (6), with peak PRL levels increasing exceeding 2-fold compared to baseline or reaching >30 ng/mL (30 μg/L) (7, 8). These results indicate appropriate reactivity.

**Figure 2.**
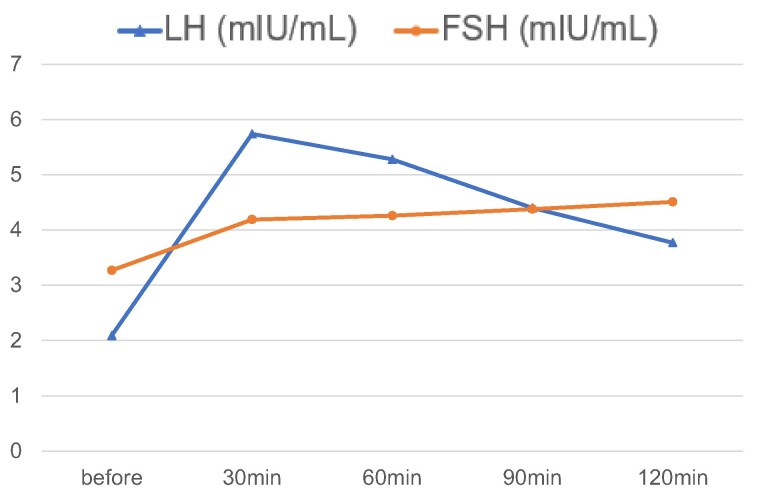
Consecutive GnRH stimulation tests. LH and FSH levels exhibited impaired responses to consecutive GnRH stimulation tests. LH and FSH data can be converted to SI units by multiplying both values by 1.

Still on day 18, the GnRH stimulation test, which involved an intravenous bolus injection of 0.1 mg of GnRH, revealed that the baseline LH and FSH levels (2.32 mIU/mL [2.32 IU/L] and 4.41 mIU/mL [4.41 IU/L], respectively) only slightly increased to their maximum levels (6.36 mIU/mL [6.36 IU/L] and 6.13 mIU/mL [6.13 IU/L], respectively) 30 and 60 minutes after the injection, respectively, revealing impaired reactivity to GnRH (Fig. 1C). In normal adult males, GnRH stimulation test shows peak LH and peak FSH levels reaching 7- to 10-fold and exceeding 2-fold increases, respectively, compared with the baseline (9). These results indicate impaired LH and FSH responsiveness to GnRH. Additionally, on day 32, in a consecutive GnRH stimulation test that involved intravenous drip infusion of 0.4 mg of GnRH for 3 hours daily for 6 consecutive days, followed by an intravenous bolus infusion of 0.1 mg of GnRH, LH, and FSH reactivity to GnRH did not improve (Fig. 2). Overall, the results revealed that the cause of hypogonadotropic hypogonadism was associated with the pituitary gland.

## Treatment

After hospitalization, the patient's daily energy intake was limited to 800 kcal (16.6 kcal/kg of ideal body weight) to reduce body weight. Additionally, he engaged in daily exercise, such as walking and cycling, totaling approximately 1 hour. Dosages of antidiabetic medications were adjusted, including increasing metformin from 1000 mg to 2000 mg and transitioning from dulaglutide to a weekly 1.0 mg subcutaneous injection of semaglutide.

## Outcome and Follow-up

**Figure 3.**
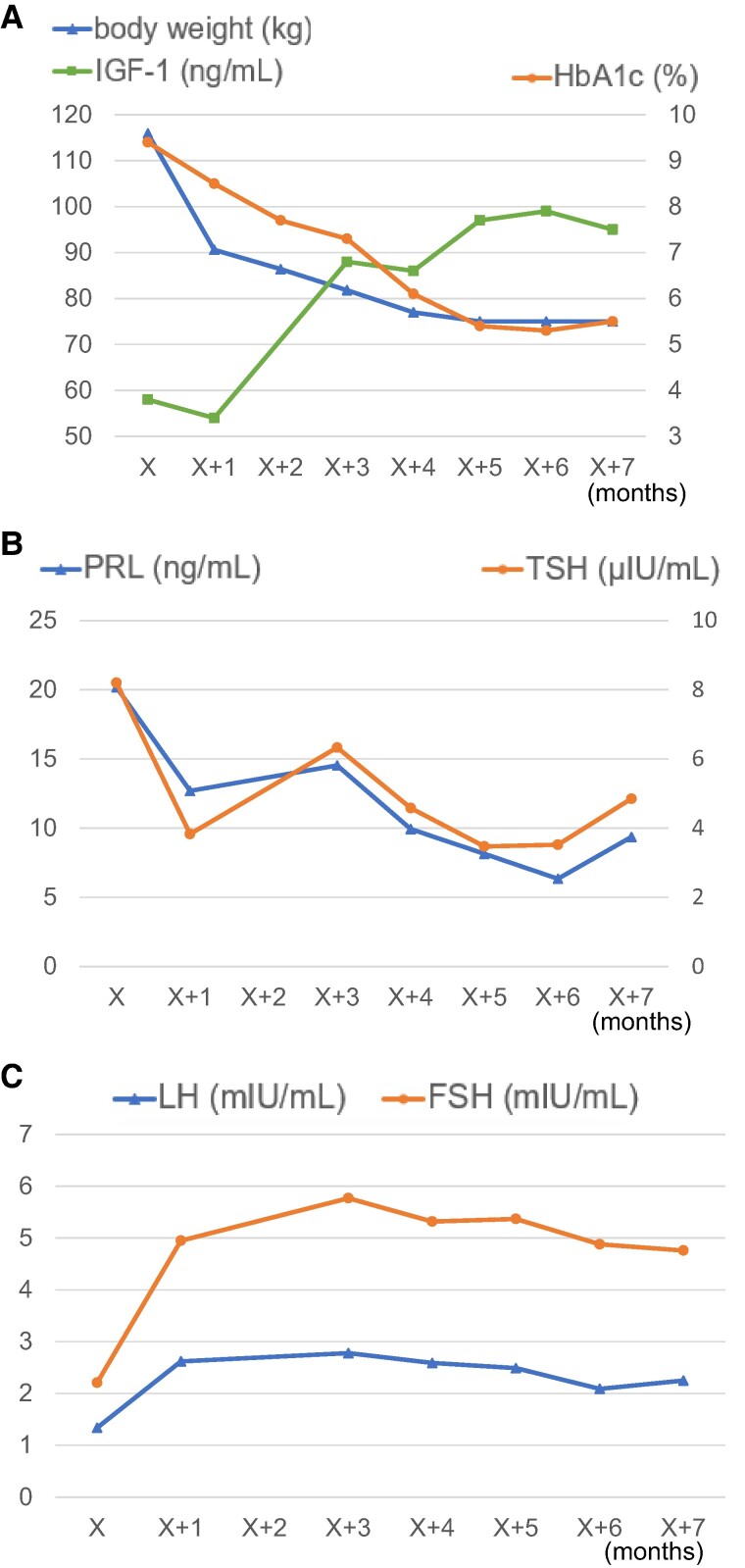
Endocrinological data postdischarge. (A) Changes in body weight, HbA1c, and IGF-1 levels. The right vertical axis represents HbA1c levels, whereas the left vertical axis represents body weight and IGF-1 levels. Gradual decreases in HbA1c levels and increases in IGF-1 levels were observed and were inversely proportional to body weight. (B) Changes in TSH and PRL levels. The right vertical axis represents TSH levels, whereas the left vertical axis represents PRL levels. TSH and PRL levels returned to within the normal ranges following weight loss. (C) Changes in LH and FSH levels. LH and FSH levels remained low following weight loss. TSH, PRL, LH, and FSH data can be converted to SI units by multiplying all values by 1. IGF-1 data can be converted to SI units by multiplying all values by 0.131.

On day 39, the patient was discharged without mobility issues or hypoxia. His weight decreased to 90.6 kg (BMI, 41.3 kg/m^2^) and HbA1c levels improved to 8.5%. Monthly assessments postdischarge, including body weight and blood testing, showed further reductions in weight and HbA1c to 75 kg (BMI, 34.2 kg/m^2^) and 5.5%, respectively, 7 months after admission (Fig. 3A). Endocrinological assessments showed a gradual increase in IGF-1 levels, reaching 95 ng/mL (12.45 nmol/L, within normal range), 7 months after admission; the increase was inversely proportional to the decrease in body weight (Fig. 3A). Additionally, elevated TSH and PRL levels decreased to within normal levels (4.85 μIU/mL [4.85 mIU/L] and 9.36 ng/mL [9.36 μg/L], respectively) (Fig. 3B). However, despite slight elevation, baseline levels of LH and FSH remained low, indicating persistent hypogonadotropic hypogonadism (Fig. 3C).

## Discussion

DS can result in several endocrine disorders that may be linked to hypothalamic GHD resulting from GHRH–GH–IGF-1 axis impairment. Here, we present a rare case of hypogonadotropic hypogonadism in a male with DS, who showed improvement in hypothalamic GHD after weight loss.

Unlike women with DS, men with DS are generally infertile because of primary hypogonadism resulting from combined Sertoli and Leydig cell dysfunction (1). Men with DS typically exhibit elevated LH and FSH levels alongside low TT and free TT levels (10). Our patient exhibited hypogonadotropic hypogonadism despite no pituitary tumors or lesions on magnetic resonance imaging. A previous study reported normal or elevated LH and FSH responses to GnRH in 6 similarly aged adult men with DS (11), suggesting our patient's impaired response indicates hypogonadotropic hypogonadism. Furthermore, although our patient achieved complete leukemia remission in infancy after chemotherapy, it is noteworthy that chemotherapy typically spares the pituitary and hypothalamic functions but can directly impact the testes or ovaries in certain cases.

Leptin, secreted by fat cells to suppress eating and maintain body weight, is increased in obese individuals. However, leptin resistance can occur in these patients, thereby suppressing the leptin receptor and inhibiting leptin signals in the hypothalamus (12). During admission, our patient was severely obese (BMI: 52.9 kg/m^2^) and had remarkably elevated leptin levels (33.1 ng/mL [33.1 μg/L]). In severely obese patients, leptin and inflammatory cytokines inhibit kisspeptin, a GnRH release stimulator, potentially causing hypogonadism by impairing the hypothalamus–gonadotroph–gonadal axis, especially in those with leptin resistance (13). After discharge, our patient's BMI and leptin levels decreased to 34.2 kg/m^2^ and 25.1 ng/mL (25.1 μg/L), representing a 35.3% and 24.2% reduction from those on admission, respectively; the decreases were partly attributed to weight loss. Reduced leptin levels following weight loss might contribute to minimal increases in baseline LH and FSH levels. However, given the typical manifestations of DS with primary hypogonadism, the relatively low postweight loss LH and FSH levels in our patient suggested persistent hypogonadotropic hypogonadism, with the exact cause remaining unknown.

Leptin influences the hypothalamus–pituitary–thyroid axis, enhancing TRH expression and promoting TSH secretion (14). In obesity, the initially elevated TSH and leptin levels usually decrease with weight loss, demonstrating a positive correlation (15). PRL levels remain stable following weight loss (16), but TRH stimulates PRL expression. Consequently, reduction of elevated leptin levels through weight loss in our patient may have lowered TSH and PRL levels, bringing them to within the ranges.

One third of patients with DS manifest with GHRH–GH–IGF-1 axis disorder, which may be attributed to hypothalamic dysfunction, resulting in low IGF-1 levels (17). In our patient, levels of IGF-1 were low, revealing hypothalamic GHD through a GHRP-2 stimulation test. Ghrelin, a stomach-derived GH-releasing peptide, stimulates food intake and GH secretion. Shiiya et al reported lower levels of plasma ghrelin in patients with obesity or type 2 diabetes mellitus compared to those in individuals with normal body weight or without diabetes mellitus (17). Date et al described a mechanism of GH secretion by ghrelin, wherein ghrelin inhibits the electrical activity of vagal afferents to GHRH neurons in the hypothalamus, leading to increased GH secretion (18). These findings align with our patient's clinical course, wherein decreased levels of IGF-1 improved and normalized following weight loss.

Glucagon-like peptide-1 receptor agonists (GLP-1RAs), such as semaglutide, are promising therapies for diabetes and obesity due to their appetite-suppressing effects and weight loss. Recent studies on GLP-1RAs and hormones such as leptin and ghrelin show that, although GLP-1RAs reduce leptin levels (19) and ghrelin stimulates native GLP-1 secretion (20), the effect on ghrelin levels is less clear. Mechanisms for leptin reduction may involve improved insulin sensitivity and increased leptin receptor expression, potentially influenced by weight loss (21, 22).

In summary, we report a case of hypogonadotropic hypogonadism in a man with DS in whom severe obesity resulted in hypothalamic GHD. Following weight loss, improvements in IGF-1, TSH, and PRL secretion were observed, while levels of LH and FSH slightly increased. These hormonal changes postweight loss may be linked to leptin and ghrelin.

## Learning Points

- Male infertility in DS is usually caused by primary hypogonadism. Although rarely, it may also be associated with hypogonadotropic hypogonadism.
- Weight loss may improve hypothalamic GHD secondary to obesity in adults with DS.
- Obesity induces leptin resistance and decreases ghrelin secretion, influencing the hypothalamus–pituitary–endocrine gland axis.

## Data Availability

Original data generated and analyzed for this case report are included in this published article.
